# Dental Phobia among Pregnant Women: Considerations for Healthcare Professionals

**DOI:** 10.1155/2020/4156165

**Published:** 2020-04-10

**Authors:** Muhammad Nazir, Muhanad Alhareky

**Affiliations:** Department of Preventive Dental Sciences, College of Dentistry Imam Abdulrahman Bin Faisal University, Dammam, Saudi Arabia

## Abstract

**Objective:**

To report the prevalence of dental phobia and associated factors among pregnant women.

**Materials and Methods:**

This cross-sectional study included pregnant women visiting hospitals in Dhahran, Khobar, and Dammam in Saudi Arabia. The Modified Dental Anxiety Scale (MDAS) was used to assess dental anxiety and phobia. The score of MDAS ranges from 5 to 25, and a cutoff value of 19 was used to identify participants with dental phobia.

**Results:**

The study analyzed data of 825 participants with mean age of 29.08 ± 5.18 years. The prevalence of dental phobia was 16.1%. About 46.9% of the sample believed that dental treatment should be avoided during pregnancy, and the importance of regular dental checkup was recognized by 16.4% of the participants. Dental phobia was associated with the perception of the health of teeth (*P* 0.004) and gums (*P* 0.016). Multiple logistic regression showed that being under the age of 30 years (OR 0.63, P 0.019) and updating knowledge about oral health (OR 0.49, *P* 0.006) were significantly associated with reduced likelihood of dental phobia. However, having bad dental experience (OR 2.13, *P* 0.001) and being in first trimester of pregnancy (OR 1.57, *P* 0.033) were significantly associated with increased odds of dental phobia.

**Conclusions:**

A considerable proportion of pregnant women reported dental phobia. The bad dental experience was associated with increased dental phobia. However, reduced likelihood of dental phobia was associated with updating oral health knowledge. Healthcare professionals may consider these factors to reduce dental phobia and improve oral health of pregnant women.

## 1. Introduction

Pregnant women are at increased risk of gingivitis, periodontitis, tooth mobility, pregnancy oral tumor, caries, and enamel erosions [1, 2]. It is known that periodontal diseases are associated with adverse pregnancy outcomes such as preterm delivery, low birth weight, and preeclampsia [3]. Periodontal diseases are also associated with diabetes, coronary heart disease, stroke, and rheumatoid arthritis [4]. Despite, there is a low utilization of oral care among pregnant women [5]. Microorganisms from the mother can colonize an infant's oral cavity [6]. Hence, inadequate oral care during pregnancy can result in poor oral health outcomes for both the mother and infants [1]. Therefore, dental care and preventive measures should be provided to pregnant women to reduce the risk of oral and systemic conditions for them and their newborns [1, 2].

Anxiety is an emotional state before the actual situation with threatening stimuli, and it may be undetectable at times. Fear is a reaction to a threat or danger, and phobia is an intense, persistent, or recurrent irrational fear that can lead to a compelling desire to avoid phobic stimuli [7]. Extreme fear of dental situation associated with uneasiness, terror, and hypertensive feelings is termed as dental phobia, a type of specific phobia, which is a clinical diagnosis as opposed to dental anxiety or fear although these terms are used interchangeably in the literature [7–9].

A large body of evidence indicates that the prevalence of dental phobia ranges from 0.9% to 12.4% in adult patients from the community or those visiting dental teaching hospitals or public dental clinics around the globe [9–14]. Dental phobia is associated with poor oral health, reduced dental attendance, and compromised quality of life [15, 16]. The patients with dental phobia were more likely to consider their dental experience more negative, report ineffective communication and lack of respect by the dentist, and receive treatment under conscious sedation [16]. Poor oral health of children is also related to dental phobia in their mothers, for example, a sample of 2–5 years of children was shown to have the highest caries experience if their mothers had dental phobia [17]. Many underlying factors influence dental phobia which has complex and multifactorial etiology [9].

A recent study showed that dentists rate dental anxiety lower than patients, and they do not effectively identify patients with dental phobia as there was no correlation between clinicians' and patients' ratings of dental anxiety in patients with dental phobia [13]. Pregnant women suffer from high burden of oral diseases, yet dental phobia, a common barrier to dental care and frequently undetected condition, is least explored. Therefore, there was a need to investigate the prevalence of dental phobia and the underlying factors in pregnant women. The identification of patients with dental phobia and awareness of its associated factors can help healthcare professionals improve the oral health of pregnant women. The study aimed to evaluate dental phobia and factors associated with it among pregnant women in Saudi Arabia.

## 2. Materials and Methods

### 2.1. Study Sample

Pregnant women visiting the King Fahad Military Medical Complex in Dhahran, King Fahad Hospital in Khobar, and Maternity and Children Hospital in Dammam were included in this study. Both Saudi and non-Saudi pregnant women who agreed to voluntary participation and provided informed consent were eligible to participate in the study. Those with psychiatric disorders, any condition impairing cognitive abilities, taking antipsychotic medication during the last 24 hours, and completely illiterate were excluded from the study. A pregnant woman was defined as illiterate if she was unable to read and sign the consent form. The study used convenience sampling for the recruitment of participants.

### 2.2. Ethical Approval

The purpose of the study was explained to eligible participants, and queries they had about research were answered. The participants were informed that their participation was voluntary, their responses were anonymous, and their refusal to participation in the study will not affect their care in the hospitals in any way. They were also informed about the anticipated time (about 15–20 minutes) for the completion of the consent form and the questionnaire. The study was conducted in accordance with the ethical principles of the Declaration of Helsinki. This investigation involves the secondary analysis of data of pregnant women from an approved project (EA 2019040) by the Scientific Research Unit (ethics committee) at the College of Dentistry, Imam Abdulrahman Bin Faisal University, Dammam, Saudi Arabia.

### 2.3. Measurement Instrument

There are several instruments used to measure dental anxiety in adults; however, Corah's Dental Anxiety Scale (DAS), Modified Dental Anxiety Scale (MDAS), and Kleinknecht's Dental Fear Survey (DFS) are common scales. In the study, dental anxiety was evaluated using the MDAS scale because it is widely preferred and used by researchers all over the world [8]. The studies have shown that the MDAS scale is consistent and reliable and has good validity [18, 19]. Also, the psychometric properties of an Arabic version of MDAS were tested in Saudi Arabia, and the scale presented adequate validity and reliability [14].

The scale includes five items that evaluate dental anxiety before going for dental treatment, waiting for dental treatment, drilling of teeth, scaling and polishing of teeth, and giving a local anesthetic injection. A 5-point Likert scale (not anxious, slightly anxious, fairly anxious, very anxious, and extremely anxious) is used for each item with 1 representing “not anxious” and 5 indicating “extremely anxious”. The score of MDAS ranges from 5 to 25, and a cutoff value of 19 was used to identify participants with dental phobia/high dental anxiety [19, 20]. Questions related to age, nationality, number of pregnancies, and trimester were included in the questionnaire. Moreover, there were questions about oral health awareness and self-perceived health of teeth and gums.

The questionnaire was reviewed to ensure its cultural adaptation in the country. Pilot testing of the questionnaire was also carried out on 30 pregnant women, and their data were excluded from the final analysis. The Arabic version of the questionnaire was used for Saudi and other Arab participants, and non-Arab participants were provided with a questionnaire in the English language. Preprinted self-complete questionnaires were distributed among study participants in the waiting areas of hospitals. Although the purpose of the study was discussed with the participants, prior information about dental anxiety was not provided to them to avoid any response bias.

### 2.4. Data Analysis

Descriptive statistics included frequencies, percentages, means, and standard deviations. A chi-square test was performed to compare oral health awareness responses between those with and without dental phobia. Similarly, the association between self-perceived health of teeth and gums with dental phobia was evaluated using the chi-square test. Multiple logistic regression analyses were performed to investigate the influence of independent variables (nationality, age, number of pregnancies, trimester, bad dental experience, dental attendance, and knowledge of oral health) on dental phobia. All statistical analyses were performed using SPSS Version 22.0 (IBM Corp. Armonk, NY, USA). The significance level was set at 5%.

## 3. Results

Data of 825 participants were included in the analysis. The prevalence of dental phobia was 16.1%. More than half the sample consisted of Saudi pregnant women (63.8%) and was under the age of 30 years (55.4%). Only 9.2% of the participants performed regular dental checkup before pregnancy, and 18.5% had a bad experience in previous dental visits (Table 1).

Forty seven percent of the participants believed that dental treatment should be avoided during pregnancy. The importance of regularly visiting the dentist was recognized by 16.4% of the participants. One-quarter of the participants (25.3%) updated oral health knowledge about pregnant women, and 72.8% were willing to receive oral health information. No significant differences were observed regarding oral health awareness of the participants with and without dental phobia except updating knowledge about the oral health of pregnant women (*P* 0.006) (Table 2).

**Figure 1:**
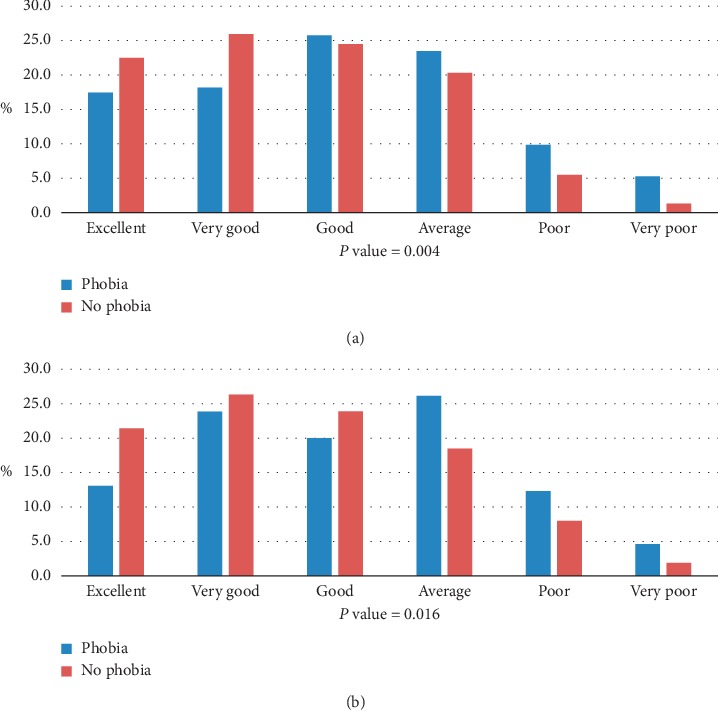
Self-perceived oral health among pregnant women with and without dental phobia. (a) Perception about health of teeth among pregnant women with and without dental phobia. (b) Perception about health of gums among pregnant women with and without dental phobia.

There was a significant association of dental phobia with the perception of the health of teeth among pregnant women (*P* 0.004). Fewer participants with dental phobia perceived the health of their teeth excellent or very good than those without dental phobia. Similar trends were observed regarding the perception of the health of gums, and dental phobia was significantly associated with the perception of the health of gums (*P* 0.016) (Figure 1).

Table 3 shows the analysis of factors that are associated with dental phobia among pregnant women. In bivariate analysis, age less than 30 years (OR 0.66, *P* 0.026), having first pregnancy (OR 0.61, *P* 0.034), performing regular dental checkup (OR 0.42, *P* 0.041), and updating knowledge about oral health (OR 0.5, *P* 0.006) were significantly associated with lower odds of dental phobia, whereas the participants who had bad dental experience were twice more likely (OR 2.01, *P* 0.041) to have dental phobia than those without dental phobia. The multiple logistic regression model showed that being in the first trimester (OR 1.54, *P* 0.041), having bad dental experience (OR 2.04, *P* 0.001) and updating oral health knowledge (OR 0.51, *P* 0.010) were significant factors associated with dental phobia.

The results of the multiple logistic regression final model (backward stepwise LR) are shown in Table 4. Being under the age of 30 years (OR 0.63, *P* 0.019) and updating oral health knowledge (OR 0.49, *P* 0.006) were significantly associated with a lower likelihood of dental phobia. However, the participants in the first trimester (OR 1.57, *P* 0.033) and those who had bad dental experience (OR 2.13, P 0.001) had significantly increased odds of dental phobia.

## 4. Discussion

The purpose of the present investigation was to evaluate the prevalence of dental phobia among pregnant women. The study found that 16.1% of the sample experienced dental phobia, and these prevalence estimates are higher than those reported in the studies of adult patients in other countries. The findings of the Adult Dental Health Survey 2009 in the UK showed that 12.4% of the population had dental phobia [16]. The prevalence of dental phobia ranged from 0.9% to 5.4% in a sample of adult Australians [9]. In the U.S, 6.82% of the study population was shown to have dental phobia [21]. The studies in India showed that 3–5% of adults seeking oral care suffered from dental phobia [10–12]. A study of Swedish adult patients indicated that the prevalence of dental phobia was 3.6% [13]. Dental phobia was reported in 2.5% of patients attending a teaching institution in Jeddah, Saudi Arabia [14]. Another study from the country indicated that the prevalence of dental phobia was 9.5% in adult Saudi patients in Riyadh [22]. However, this comparison shows dental phobia is more common in pregnant women than adult population. Nevertheless, it should be noted that high risk of dental phobia in pregnant women may be related to the female gender [16, 21].

In a previous study, the participants with dental phobia considered their dental experience more negative compared with those without phobia [16]. The negative dental experience was identified as one of the sources of dental anxiety in a sample of patients in dental practice in the US [21]. Similarly, health profession students in Saudi Arabia with a bad dental experience demonstrated higher dental anxiety than those with a positive experience [23]. Similar findings were reported in a study from India [24]. Distressing experience in the past dental visits is impacted in the increased risk of dental phobia because extreme helplessness and embarrassment during dental treatment and the dentist's lack of understanding were reported the most important experiences that affect dental phobia [25]. Analysis of past dental experience in our study showed that pregnant women with bad experience were 2.13 times more likely to have dental phobia than those without such experience. Thus, the results of the present study emphasize the importance of providing state-of-the-art dental care and positive dental experience to potentially reduce dental phobia in pregnant women.

Women in the first trimester demonstrate more anxiety and responsiveness to chemosensory signals than women in the second and third trimesters [26]. This pattern shows close similarity to what was observed in the present study, with women in the first trimester who were 1.57 times more likely to report dental phobia than those in the second and third trimesters. Pregnant women under the age of 30 years were 0.63 times less likely to have dental phobia than those who were equal or more than 30 years in our study. This finding highlights the need for better management of pregnant women with dental phobia in older age. Similar to our findings, Heidari et al. reported that out of 1367 dental phobia participants, 28.7% were below 34 years and 42% were between 35 and 55 years of age [16]. The results of a study by Humphris et al. showed that 14.3% of the participants in the 18–29 year age group had dental phobia compared with 17.3% of participants in the 30–39 year age group. Nevertheless, the authors reported that the participants aged 18–39 years had dental anxiety four times higher than those who were 60 years of age or over [19]. Similarly, White et al. reported that one unit increase in the age of participants was associated with 0.08 unit reduction in the MDAS score [21]. These inconsistencies regarding the association of age with dental phobia/anxiety should be further investigated using large data from cohort studies.

A small percentage of participants in our study recognized the importance of regular dental attendance, and many believed in avoiding dental treatment during pregnancy. The pregnant women who updated their oral health knowledge about pregnancy had a significantly reduced likelihood of having dental phobia. It is known that the dissemination of oral health information improves health literacy [27], and participants with low oral health literacy were shown to have significantly increased periodontal disease and tooth loss and reduced filled teeth than those with adequate oral health literacy [28]. Therefore, improved oral health information can lead to effective communication, enhanced patient adherence, prompt access to care, improved self-management skills, and positive outcomes of oral care [27, 28]. Our study data provide evidence to support the provision of oral health information to reduce dental phobia. Dental professionals and family members should encourage pregnant women to seek oral care and perform regular dental visits because their advice regarding dental attendance is well received by pregnant women who passively obtain oral health information disseminated through mass media and social environment [29].

Dental phobia, lack of dental awareness, financial barriers, difficult access to dental service, misunderstandings/misconceptions about dental care during pregnancy, and low priority for oral health are some of the reasons for reduced utilization of oral care in pregnant women [16, 29]. Awareness about oral health should be raised so that pregnant women can receive the needed oral care and improve their oral health as increased dental anxiety can lower their oral health-related quality of life [30]. They should be encouraged to receive oral care during the second trimester of pregnancy which is considered safe for the provision of dental treatment including diagnostic radiography [2]. Developing collaboration among medical and dental healthcare providers and providing them updated oral health information can result in increased oral health knowledge and optimal oral health in pregnant women.

The present study should be considered in light of its limitations. The data were collected from a nonprobability sample of pregnant women who conveniently participated in the study. In previous research, Armfield argued that the MDAS scale has some limitations with regards to its theoretical framework [9]. The study included a fairly large sample of pregnant women from public tertiary care hospitals from three cities in the eastern region of Saudi Arabia; however, this limits the generalizability of study findings to pregnant women visiting public health centers and private clinics and hospitals in the province. A cross-sectional study design also is subject to bias due to underrepresentation or overrepresentation of responses.

## 5. Conclusions

The study showed that dental phobia was common among pregnant women. Many women believed that dental treatment should be avoided during pregnancy, and few recognized the importance of visiting the dentist regularly. Pregnant women under the age of 30 years and those who updated their oral health knowledge were less likely to experience dental phobia. Bad dental experience and being in the first trimester of pregnancy increase the likelihood of dental phobia.

Dental professionals should ensure high-quality dental care to avoid bad dental experience and prevent dental phobia. Pregnant women in the first trimester and in older age groups should be given particular attention to achieve a reduction in dental phobia. Medical, dental, and other healthcare providers should play their role in improving oral health knowledge of pregnant women.

## Figures and Tables

**Table 1:** Distribution of study variables among pregnant women.

Study variables	*N* = 825	95% CI^*∗*^
Dental phobia	133 (16.1)	(13.7, 18.8)

Nationality		
Saudi	526 (63.8)	(60.5, 67)
Non-Saudi	299 (36.2)	(33, 39.6)

Age		
<30	457 (55.4)	(52, 58.8)
≥30 years	368 (44.6)	(41.2, 48)

Number of pregnancies		
First pregnancy	216 (26.2)	(23.2, 29.2)
Second or more pregnancies	609 (73.8)	(70.8, 76.8)

Trimester of pregnancy		
First trimester	213 (25.8)	(22.8, 28.8)
Second and third trimesters	612 (74.2)	(71.2, 77.2)

Had bad experience in previous dental visit (s)	153 (18.5)	(15.9, 21.2)

Visited a dentist in the last year	421 (51)	(47.6, 54.4)

Performed regular checkup before pregnancy	76 (9.2)	(7.2, 11.2)

^*∗*^CI = confidence interval.

**Table 2:** Oral health awareness among pregnant women with and without dental phobia.

Study variables	*N* = 825	Pregnant women with dental phobia N (%)	Pregnant women without dental phobia N (%)	*P* value
Recognized the importance of regular dental checkup during pregnancy.	135 (16.4)	19 (14.1)	116 (85.9)	0.479
Believed that dental treatment should be avoided during pregnancy.	387 (46.9)	54 (14)	333 (86)	0.111
Believed that tooth and gum problems could affect the outcomes of pregnancy.	245 (29.7)	39 (15.9)	206 (84.1)	0.918
Recognized the importance of good oral health for infant.	171 (20.7)	34 (19.9)	137 (80.1)	0.133
Believed that pregnancy increases the risk of gum bleeding, swelling, or redness.	147 (17.8)	16 (10.9)	131 (89.1)	0.057
Updated knowledge about the oral health of pregnant women.	209 (25.3)	21 (10)	188 (90)	0.006^*∗*^
Wanted to receive information about the oral health of pregnant women.	601 (72.8)	95 (15.8)	506 (84.2)	0.688

**Table 3:** Association between various factors and dental phobia in pregnant women.

Study variables	Unadjusted odds ratio (OR)	*P* value	Adjusted odds ratio (OR)	*P* value
Nationality	1.01 (0.68, 1.48)	0.968	0.91 (0.60, 1.36)	0.626
Saudi				
Non-Saudi				

Age	0.66 (0.45, 0.95)	0.026^*∗*^	0.67 (0.45, 1.01)	0.054
<30				
≥30 years				

Number of previous pregnancies	0.61 (0.38, 0.97)	0.034^*∗*^	0.77 (0.46, 1.27)	0.300
First pregnancy				
Second or more pregnancies				

Trimester pregnancy	1.47 (0.98, 2.19)	0.061	1.54 (1.02, 2.33)	0.041^*∗*^
First trimester				
Second and third trimesters				

Had a bad experience in previous dental visit (s)	2.01 (1.31, 3.07)	0.001^*∗*^	2.04 (1.31, 3.18)	0.001^*∗*^
Visited a dentist in the last year	0.73 (0.5, 1.06)	0.093	0.71 (0.49, 1.05)	0.088
Performed regular checkup before pregnancy	0.42 (0.18, 0.99)	0.041^*∗*^	0.53 (0.22, 1.27)	0.156
Updated knowledge about the oral health of pregnant women	0.5 (0.31, 0.82)	0.006^*∗*^	0.51 (0.31, 0.85)	0.010^*∗*^

^*∗*^Statistically significant.

**Table 4:** Multiple logistic regression final model (backward stepwise LR): association between various factors and dental phobia in pregnant women.

Study variables	Adjusted odds ratio (OR)	*P* value
Age	0.63 (0.43, 0.93)	0.019^*∗*^
<30		
≥30 years		

Trimester pregnancy	1.57 (1.04, 2.38)	0.033^*∗*^
First trimester		
Second and third trimesters		

Had a bad experience in previous dental visit (s)	2.13 (1.37, 3.29)	0.001^*∗*^
Visited a dentist in the last year	0.69 (0.47, 1.02)	0.059
Updated knowledge about the oral health of pregnant women	0.49 (0.3, 0.82)	0.006^*∗*^

Adjusted for age, nationality, trimester of pregnancy, bad dental experience, dental attendance, regular dental visits, and updating oral health knowledge. ^*∗*^Statistically significant.

## Data Availability

The SPSS data file of this study is available from the corresponding author upon request.
